# Arachnids (Araneae, Opiliones) from grass stand and forest litter in the Urals, Russia

**DOI:** 10.3897/BDJ.8.e55242

**Published:** 2020-10-08

**Authors:** Alexey Nesterkov, Maxim Zolotarev, Elena Belskaya, Tatyana Tuneva

**Affiliations:** 1 Institute of Plant and Animal Ecology (IPAE), Yekaterinburg, Russia Institute of Plant and Animal Ecology (IPAE) Yekaterinburg Russia

**Keywords:** Occurrence, diversity, abundance, seasonal dynamics, interannual dynamics, sex ratio, age-sex composition, life stage

## Abstract

**Background:**

Since the late 1980s, long-term monitoring of various components of natural ecosystems under conditions of industrial pollution has been carried out in the Central Urals. In the mid-2000s, similar programmes were started in the Southern Urals. As a part of these monitoring programmes, the data on invertebrates in different types of biotopes, collected with different methods and in a different time intervals, continue to be gathered. Amongst the most well-studied groups of invertebrates are spiders and harvestmen whose communities are a convenient indicator of the environment. The data collected through these monitoring programmes can also be used to study natural local biodiversity.

**New information:**

The dataset, presented here, includes information from a long-term monitoring programme for Araneae and Opiliones that inhabit grass stands of secondary dry meadows and litter of spruce-fir, aspen-birch and pine-birch forests in the Central and Southern Urals. The dataset (available from the GBIF network at https://www.gbif.org/dataset/e170dbd1-a67f-4514-841c-5296b290ca90) describes the assemblage structure of spiders and harvestmen (list of species and their abundance), age-sex composition and seasonal and inter-annual dynamics for two large areas in the southern taiga zone of the Ural Mountains. The dataset includes 1,351 samples, which correspond to 5,462 occurrences identified during 2004–2009, 2013 and 2018. In total, we collected 10,433 specimens, representing 178 species (36% of arachnofauna of the Urals), 115 genera (54%) and 23 families (100%). Most of the data (4,939 of 5,462 occurrences, 90%) were collected in the western macro-slope of the Ural Mountains (European part of Russia), the rest in the eastern macro-slope (Asian part). All represented data were sampled in industrially undisturbed areas and are used as a local reference for ecotoxicological monitoring. The dataset provides new useful information for recording the state of biodiversity for the Central and Southern Urals and contributes to the study of biodiversity conservation.

## Introduction

The arachnids are a widespread group of invertebrates; almost all Araneae and many Opiliones are obligate predators. It was shown that spiders can be used as indicators of local diversity (Willett 2001). All parameters (abundance, diversity, evenness and species richness) of arachnid communities demonstrate a close relationship with the structure of their habitats (Rubio et al. 2008). This makes arachnids a good tool for assessing the components of diversity on a local spatial scale (Rodriguez-Artigas et al. 2016).

The fauna of spiders and harvestmen in the Central and Southern Urals is currently one of the most studied in Russia. At the present time, the fauna of Araneae of the Urals includes 485 species belonging to 202 genera from 23 families (Esyunin 2015) and the fauna of Opiliones includes 10 species from 10 genera of two families (Farzalieva and Esyunin 1999). Local diversity ratings of the fauna are lower for Araneae: there are 235 species and 127 genera for the Central Urals and 180 species and 112 genera for the Southern Urals (Esyunin 2015). For Opiliones, local diversity is almost no different from the regional: there are 10 species and 10 genera in the Central Urals and eight species and eight genera in the Southern Urals (Farzalieva and Esyunin 1999). The first special work devoted to the fauna of the spiders of the Urals contained information on 86 species collected mainly in the vicinity of Yekaterinburg (Kharitonov 1923). Subsequently, this same researcher published a catalogue with more than 200 species of spiders recorded for the Urals and Cisurals (Kharitonov 1932, Kharitonov 1936). In the second half of the twentieth century, intensive multi-year studies were carried out for various territories of the Central (Azheganova and Glukhov 1981, Esyunin 1991, Pakhorukov et al. 1995) and Southern (Olshvang and Malozyomov 1987, Polyanin and Pakhorukov 1988, Esyunin and Polyanin 1990) Urals. As a result of this work, a catalogue was published with a summary of all the information available at that time on the fauna of the spiders of the Urals and Cisurals (Esyunin and Efimik 1996). The global fauna of harvestmen was poorly studied until the end of the 1970s, when the number of studies began to gradually increase (Kury 2012). Harvestmen fauna of the USSR includes 74 species in the mid-1930s (Redikortsev 1936) and 110 species in the late 1970s (Staręga 1978). The last paper presents the first data on the species composition of harvestmen in the Urals region (specified as a part of Western Siberia). By this time, the fauna of the European part of the USSR (58 species) has been the most well-studied (Chevrizov 1979). A comprehensive review of harvestmen of the Urals (10 species) is given later in the catalogue of local fauna (Farzalieva and Esyunin 1999).

The presented dataset for the Central Urals contains 166 species (of which 159 species (68% of total regional fauna) are spiders and seven (70%) are harvestmen) and for the Southern Urals, it contains 55 species (53 (29%) and two (25%), respectively). Poor level of knowledge of the fauna of the Southern Urals is caused by the limited extent of monitoring (currently only one year). The family with the greatest number of species and genera is Linyphiidae (50% and 56%, respectively). In the temperate climatic part of the Urals, local arachnid fauna are comparable in terms of the ratio of families with the largest number of species (Esyunin 2015).

Spider fauna of the Urals has a number of distinctive features. Firstly, it can be characterised as poor: the diversity is lower than that of the fauna of the adjacent plains (both East European and West Siberian (Esyunin 2015)), as well as of the neighbouring mountainous countries. For example, 1110 species are known for the arachnofauna of the Caucasus (Otto 2019) and 614 species for the Republic of Tyva, South Siberia (Marusik et al. 2000). Secondly, the fauna has an extremely low endemicity. This, along with a low diversity, indicates the allochthonous character of the Ural fauna of spiders and the young age of its modern composition (Esyunin 2015).

## Project description

### Title

Biota of contaminated areas under high pollution and during the reduction of industrial emissions

### Personnel

Evgeny Vorobeichik

### Study area description

**Figure 1.**
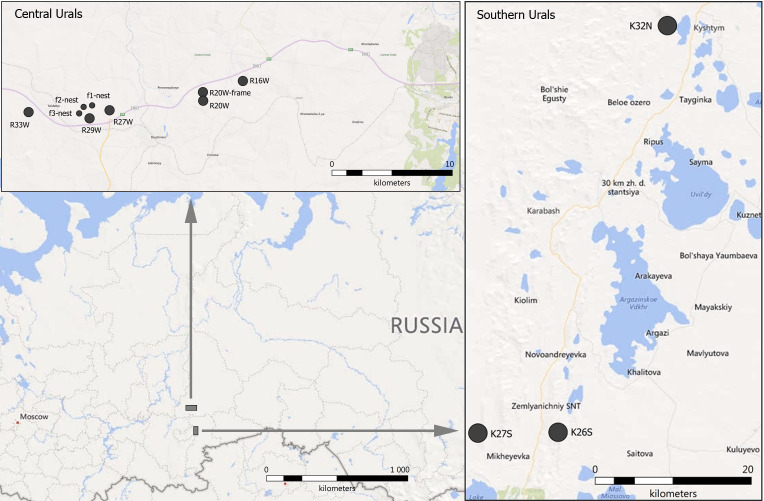
Location of the sampling plots in the Central and the Southern Urals (data from SASPlanet).

The Ural Mountains are a north-south orientated mountain system in the Urals, located between the East European and West Siberian plains (Fig. 1). Both studied areas in the Central and Southern Urals are located in the lowest part of uplands (300–400 m above sea level) in the southern taiga zone. In the Central Urals, the prevailing forest types are primary spruce-fir and secondary aspen-birch forests; in the Southern Urals, the prevailing forest type is pine-birch forest. Annual temperature and precipitation averages are similar between the two study areas: 1.7°C and 575 mm for the Central Urals, 1.8°C and 556 mm for the Southern Urals.

## Sampling methods

### Study extent

The study was conducted in the southern taiga zone of the Central and Southern Urals, Russia, in the lowest part of the uplands (300–400 m above sea level). A total of twelve sampling plots (= locationID) were established across three types of biotopes: primary spruce-fir (four plots), secondary aspen-birch forests (two plots), pine-birch forest (three plots) and secondary upland meadows (three plots).

All the represented sampling schemes refer to different ongoing long-term monitoring projects, consolidated by the same research objects. At the present time, sampling with the biocenometer was carried out in the Central Urals on 21 June 2006 – 02 September, 2006; 25 June 2007 – 29 August 2007; 17 June 2008 – 28 August 2008. Line-designed pitfall trapping in the Central Urals was carried out on 12 May 2004 – 24 August 2004; 10 June 2009 – 08 September 2009; 14 June 2013 – 02 September 2013; in the Southern Urals on 28 May 2009 – 01 September 2009. Matrix-designed pitfall trapping was carried out in the Central Urals on 04 May 2005 – 16 August 2005; 25 May 2018 – 21 August 2018.

### Sampling description

Sampling of meadow grass stand invertebrates was completed using a biocenometer. Samples were collected at three permanent free-form sampling plots (approximately 2500 m^2^ in size) that were positioned at a distance of 100–300 m from each other in the lower parts of the secondary upland meadows created through forest clear-cutting more than 60 years ago (Table 1). Sampling effort (time interval for collecting one sample) was approximately 25 minutes. All samples were collected no closer than 10 m from the forest edge. The points for installing the biocenometer were chosen randomly, but at intervals of no less than 5 m. The sampling procedure was carried out from 09:00 to 21:00 h local time. The sampling plots were examined on the same day. Morning-, midday- and evening-time-collected samples were available for each investigated plot. Sampling was timed to the second half of every summer month (10 samples per plot) from 2006 to 2008.

**Figure 2.**
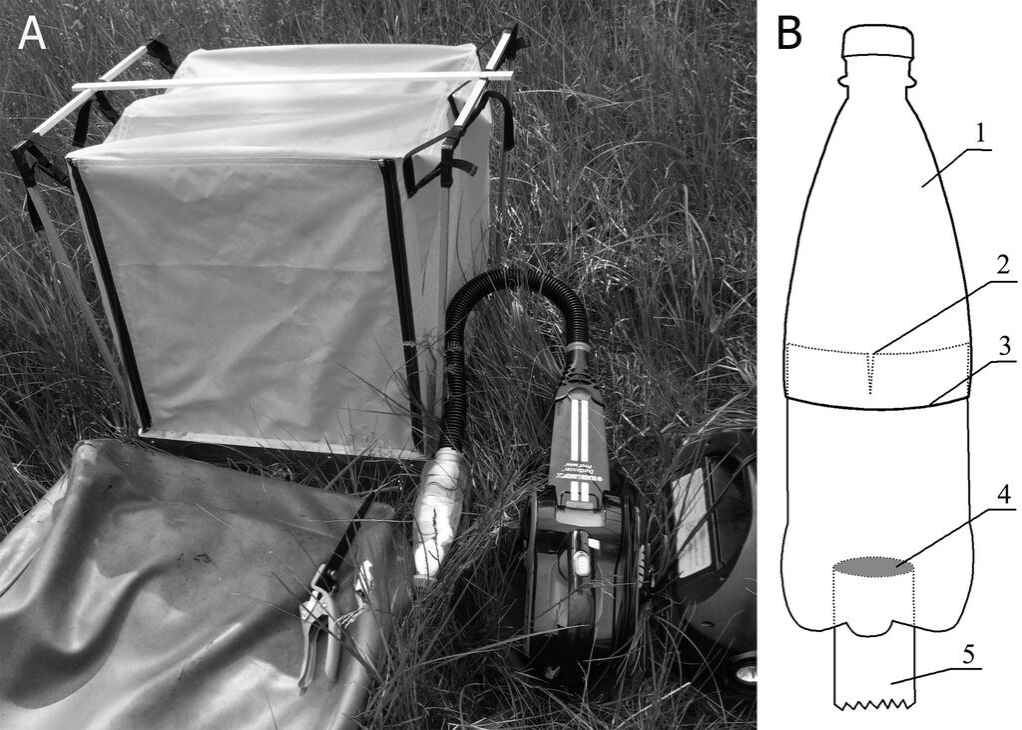
Appearance of the biocenometer (A) and a circuit of an original sampling header (B) (1 – detachable part of the bottle, 2 – longitudinal cuts, 3 –perpendicular cut, 4 – membrane of nylon gauze, 5 – air intake pipe)

Sampling was performed by using a modified biocenometer consisting of a bottom (metal frame 50×50 cm) hermetically connected to a cube-shaped covering of a dense cloth (Fig. 2). One of the lateral sides of covering was sewn from nylon gauze (mesh diameter 0.25 mm) and used as a light screen to attract invertebrates with positive photokinesis. The opposite-to-screen side of the covering contains an aperture with an inlet valve for the researcher. Invertebrates were collected with a suction sampler from a light screen and inner surfaces of the biocenometer until the new targets stopped appearing. All the plants that got inside were also processed with a suction sampler (to gather invertebrates), cut with scissors at ground level and taken away for a manual check for hidden invertebrates. Then the biocenometer was turned over and its inner surface and seams were examined, as well as the soil surface and bases of the plant stems. All detected invertebrates were devitalised by ethyl acetate and preserved in 70% alcohol.

**Figure 3.**
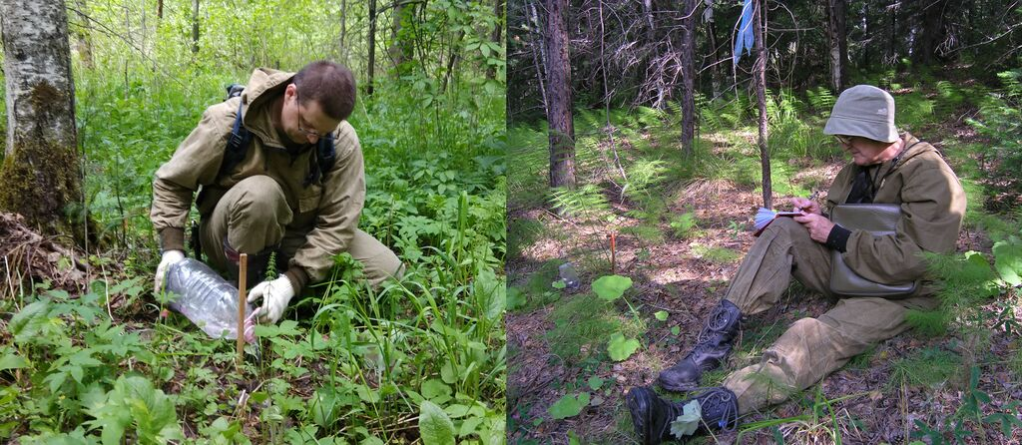
Mounting of a pitfall trap.

Pitfall trapping was carried out in biotopes most typical for the studied areas: primary spruce-fir forest, secondary aspen-birch forest and pine-birch forest (Fig. 3). Sampling plots were founded in sites with the lowest degree of degradation of woody vegetation (Table 1). Sampling of forest litter invertebrates was conducted with two general schemes. The line-designed scheme (used for regular periodic accounting of forest litter invertebrates) includes five pitfall traps per trapping line, with a spacing of 3 m and three lines per sampling plot no closer than 100 m from each other (a total of eight plots, each approximately 2,400 m² in size) were examined both in spruce-fir (three plots) and aspen-birch (five plots) forests. The matrix-designed scheme (used to study spatial heterogeneity of forest litter invertebrates in different years) includes a 7×7 matrix of pitfall traps with 10 m spacing on a single square-form sampling plot (3,600 m²) in a spruce-fir forest. Pitfall traps of the same type (plastic glasses, diameter 8.5 cm, 3% acetic acid solution as a fixative) were used in both schemes. All plots and locations of every trap were permanent throughout the study. Sampling was conducted in twotime-sets; May–June and August–September (which is timed to the peak abundance of spring-summer and summer-autumn species) in 2004, 2009 and 2013 (for line-designed trapping) and in 2005 and 2018 (for matrix-designed trapping). The traps were emptied once per 3 to 6 days; all collected invertebrates were preserved in 70% alcohol.

### Quality control

A total of more than 10400 individuals of spiders and harvestmen were collected. All specimens were wet-preserved in 70% alcohol and stored in the depository of the Laboratory for Population and Community Ecotoxicology of the Institute of Plant and Animal Ecology, Ural Branch, Russian Academy of Sciences (IPAE UB RAS). Most of the adult specimens were identified to species (except for those severely damaged during the sampling). Species identification was also carried out on juvenile specimens when there was no doubt about their identity. Identification of species was performed by a permanent team of researchers (IPAE UB RAS) using Nentwig et al. 2019 for spiders and identification keys of Farzalieva and Esyunin 1999 for harvestmen, as well as some additional monographs (Chevrizov 1979). Identification quality was cross-checked by Professor Sergey L. Esyunin from the Department of Zoology of Perm State University. Spider nomenclature follows the World Spider Catalogue (2020); harvestmen nomenclature follows de Jong et al. (2014) and the local catalogue of Ural’s fauna (Farzalieva and Esyunin 1999).

## Geographic coverage

### Description

The studied areas are located in the southern taiga zone of the Central and Southern Urals. The polygon at the Central Urals is located 60–70 km westbound from Yekaterinburg in primary spruce-fir and secondary aspen-birch forests with secondary upland meadows created through clear-cutting. At the Southern Urals, two polygons are located 10 and 60 km NE from Miass, in pine-birch forest.

### Coordinates

55.1 and 56.868 Latitude; 56.82 and 60.793 Longitude.

## Taxonomic coverage

### Description

The dataset contains all of the information obtained during the sampling for the Arachnida class (including Araneae and Opiliones orders). General taxonomic coverage is one Phylum, two Orders, 23 Families, 115 Genera and 178 Species (Nesterkov et al. 2020).

### Taxa included

**Table:** 

Rank	Scientific Name	Common Name
class	Arachnida	arachnids
order	Araneae	spiders
order	Opiliones	harvestmen

## Traits coverage

### Data coverage of traits

PLEASE FILL IN TRAIT INFORMATION HERE

## Temporal coverage

### Notes

At the present time, the following periods are covered: 12 May 2004 – 08 September 2009; 14 June 2013 – 02 September 2013; 24 May 2018 – 21 August 2018.

## Collection data

### Collection name

Паукообразные_СУМЗ_КМЗ

### Specimen preservation method

alcohol

## Usage rights

### Use license

Creative Commons Public Domain Waiver (CC-Zero)

### IP rights notes

This work is licensed under a Creative Commons Attribution (CC-BY) 4.0 License.

## Data resources

### Data package title

Arachnids (Aranei, Opiliones) from grass stand and forest litter in the Urals, Russia

### Resource link

https://doi.org/10.15468/y865v3

### Alternative identifiers

https://www.gbif.org/dataset/e170dbd1-a67f-4514-841c-5296b290ca90; http://gbif.ru:8080/ipt/resource?r=arachnids_ural

### Number of data sets

1

### Data set 1.

#### Data set name

Arachnids (Aranei, Opiliones) from grass stand and forest litter in the Urals, Russia

#### Data format

Darwin Core

#### Number of columns

38

#### Description

The dataset describes the quantitative and qualitative structure of arachnids, age-sex composition and seasonal and inter-annual dynamics for two large areas in the southern taiga zone of the Ural mountains. Arachnids were sampled with three general schemes, which allowed the coverage of a wide range of habitats: inhabitants of grass stand were collected using biocenometer (three sampling plots (= locationID) in total), inhabitants of forest litter were collected using line-designed (eight plots) and matrix-designed pitfall trapping (one plot). The dataset includes 1351 samples (= sampling events), which corresponded to 5462 occurrences identified during 2004–2009, 2013 and 2018. In total, we collected 10433 specimens, representing 178 species (36% of arachnofauna of the Urals), 115 genera (54%) and 23 families (100%). Only samples that contained arachnids (occurrenceStatus = present) have been provided. The dataset represents the new data useful for recording the state of biodiversity of a region and contributes to the study of biodiversity conservation.

**Data set 1.** 

Column label	Column description
eventID	An identifier for the set of information associated with an Event (something that occurs at a place and time). May be a global unique identifier or an identifier specific to the dataset.
occurrenceID	An identifier for the Occurrence (as opposed to a particular digital record of the occurrence).
basisOfRecord	The specific nature of the data record.
specificEpithet	The name of the first or species epithet of the scientificName.
organismQuantity	A number or enumeration value for the quantity of organisms.
organismQuantityType	The type of quantification system used for the quantity of organisms.
scientificName	The full scientific name, with authorship and date information, if known.
kingdom	The full scientific name of the kingdom in which the taxon is classified.
phylum	The full scientific name of the phylum or division in which the taxon is classified.
class	The full scientific name of the class in which the taxon is classified.
order	The full scientific name of the order in which the taxon is classified.
family	The full scientific name of the family in which the taxon is classified.
genus	The full scientific name of the genus in which the taxon is classified.
taxonRank	The taxonomic rank of the most specific name in the scientificName.
sex	The sex of the biological individual(s) represented in the Occurrence.
lifeStage	The age class or life stage of the biological individual(s) at the time the Occurrence was recorded.
occurrenceRemarks	Comments or notes about the Occurrence.
recordedBy	A list (concatenated and separated) of names of people, groups or organisations responsible for recording the original Occurrence.
identifiedBy	A list (concatenated and separated) of names of people, groups or organisations who assigned the Taxon to the subject.
samplingProtocol	The name of, reference to, or description of the method or protocol used during an Event.
samplingEffort	The amount of effort expended during an Event.
sampleSizeValue	A numeric value for a measurement of the size (time duration, length, area or volume) of a sample in a sampling event.
sampleSizeUnit	The unit of measurement of the size (time duration, length, area or volume) of a sample in a sampling event.
eventDate	The date-time or interval during which an Event occurred.
habitat	A category or description of the habitat in which the Event occurred.
year	The four-digit year in which the Event occurred, according to the Common Era Calendar.
month	The ordinal month in which the Event occurred.
country	The name of the country or major administrative unit in which the Location occurs.
countryCode	The standard code for the country in which the Location occurs.
stateProvince	The specific description of the place.
municipality	The full, unabbreviated name of the next smaller administrative region than county (city, municipality etc.) in which the Location occurs. Do not use this term for a nearby named place that does not contain the actual location.
locality	The specific description of the place. Less specific geographic information can be provided in other geographic terms (higherGeography, continent, country, stateProvince, county, municipality, waterBody, island, islandGroup). This term may contain information modified from the original to correct perceived errors or standardise the description.
locationID	An identifier for the set of location information (data associated with dcterms:Location).
decimalLatitude	The geographic latitude (in decimal degrees, using the spatial reference system given in geodeticDatum) of the geographic centre of a Location.
decimalLongitude	The geographic longitude (in decimal degrees, using the spatial reference system given in geodeticDatum) of the geographic centre of a Location.
geodeticDatum	The ellipsoid, geodetic datum or spatial reference system (SRS) upon which the geographic coordinates given in decimalLatitude and decimalLongitude are based.
coordinateUncertaintyInMetres	The horizontal distance (in metres) from the given decimalLatitude and decimalLongitude describing the smallest circle containing the whole of the Location. Leave the value empty if the uncertainty is unknown, cannot be estimated or is not applicable (because there are no coordinates). Zero is not a valid value for this term.
ownerInstitutionCode	The name (or acronym) in use by the institution having ownership of the object(s) or information referred to in the record.

## Additional information

### Discussion

We collected a total of 10,433 specimens of arachnids (7,527 spiders and 2,906 harvestmen, Table 2), of which 9,659 specimens were from the Central Urals (6,767 and 2,892, respectively) and 774 specimens from the Southern Urals (760 and 14). In the latter case, sampling of invertebrates was carried out only in 2009, which explains a significantly lower number of specimens.

In the Central Urals, three types of biotopes were investigated: meadow grass stand (502 spiders and 250 harvestmen), spruce-fir (4,962 and 2,129, respectively) and aspen-birch (1,303 and 513) forest litter. In the grass stand, 21 families of arachnids were detected; the greatest abundance and species richness were revealed in Phalangiidae (31% of specimens, 7% of species), Linyphiidae (28% and 40%, respectively), Lycosidae (10% and 9%) and Salticidae (7% and 7%). In the spruce-fir forest litter (17 families), the most abundant and rich with species were Linyphiidae (65% of specimens, 61% of species), Phalangiidae (18% and 5%), Nemastomatidae (12% and 1%) and Lycosidae (2% and 10%). In the aspen-birch forest litter (14 families), the dominant families were the same: Lycosidae (34% and 10%), Linyphiidae (33% and 64%), Phalangiidae (23% and 5%) and Nemastomatidae (5% and 1%).

In the Southern Urals, sampling was carried out only in the prevailing biotope, the pine-birch forest litter (760 spiders and 14 harvestmen). A total of 16 families were revealed; greatest abundance and species richness were found in Lycosidae (40% of specimens, 11% of species), Linyphiidae (28% and 51%) and Thomisidae (13% and 7%). The family with the greatest number of species and genera is Linyphiidae, which is typical for arachnofauna of the climatically-temperate part of the Urals (Esyunin 2015).

It is interesting that the family Oxyopidae is represented only by juvenile specimens of *Oxyopes ramosus* (Martini & Goeze, 1778) (Table 2). This is a xerophilous species, preferring open biotopes; in high latitudes, it is found in clearings, meadows and in the mountain-tundra belt. Based on our observations, adult individuals of this species are found mainly in shrubs (tamnobiontous); in the grass stand, these spiders are few and immature individuals are prevailing. Perhaps this is due to the peculiarities of the population structure at the northern boundary of the distribution of the species.

Age-sex composition is an important characteristic of the state of natural communities. For spiders, adult individuals predominate (Table 3), which is apparently related to the periodisation of pitfall trapping, attributed specifically to the peaks of abundance of the adults (May-June and August-September). Moreover, many species of the spider communities of temperate latitudes have a two-year life cycle (Schaefer 1987). For harvestmen, the ratio of age groups is also typical (predominance of juveniles) and corresponds to a one-year life cycle (Belozerov 2012).

With reference to the sex ratio of both spiders and harvestmen in temperate latitudes, the predominance of females is characteristic throughout the summer season (Huhta 1965, Schaefer 1987). However, this tendency manifests itself only for the meadow grass stand communities, while the forest litter is featured with the prevalence of males (Table 3). The reason, apparently, is the difference in sampling methods. For meadows, we used a biocenometer that provides a relatively complete registration of invertebrates; this allowed us to reveal the most typical ratio of sexes (Huhta 1965, Schaefer 1987). Communities of the forest litter were studied using pitfall traps, which allow recording the activity density. In some species of arachnids, males are more active (Topping and Sunderland 1992), which explains the higher abundance values. In addition, some species of spiders are more able than others to get out of traps (Topping 1993); perhaps this can also affect the numbers of male and female spiders caught.

Amongst the interesting finds of species, it is important to point out *Sintula corniger* (Blackwall, 1856) of Linyphiidae. This is a rare (widely distributed, but not numerous everywhere) species with a trans-European nemoral distribution area (from Great Britain and France to the Urals, from Fennoscandia to Romania and Azerbaijan (Nentwig et al. 2019, World Spider Catalogue 2020)). The Ural mountain range is the easternmost distribution boundary of this species, due to the north-eastern limit of distribution of the nemoral flora. The species was found in an aspen-birch forest, where an element of nemoral flora (small-leaved linden, *Tilia cordata*) is present in the understorey. Two adult male specimens were caught in pitfall traps in May 2004. This is the first find of this species in the Central Urals.

## Figures and Tables

**Table 1.** Coordinates of the sampling plots (300–400 m above sea level)

Sampling Protocol	Habitat	Plot (=locationID)	Trap-line	Latitude б Longitude
biocenometer	meadow grass stand	f1-nest	–	56.801359, 59.427505
		f2-nest	–	56.802545, 59.428337
		f3-nest	–	56.802794, 59.429678
line-designed pitfall trapping	pine-birch forest litter	K26S	1	55.236278, 60.202972
			2	55.236972, 60.203028
			3	55.237528, 60.203917
		K27S	1	55.224444, 60.124111
			2	55.231333, 60.123611
			3	55.232111, 60.123972
		K32N	1	55.712611, 60.466833
			2	55.713083, 60.466917
			3	55.713083, 60.467167
	aspen-birch forest litter	R16W	1	56.831278, 59.642500
			2	56.831556, 59.636694
			3	56.832111, 59.641028
		R27W	1	56.805528, 59.465056
			2	56.806111, 59.466167
			3	56.807000, 59.466778
	spruce-fir forest litter	R20W	1	56.816283, 59.575745
			2	56.816845, 59.575736
			3	56.817134, 59.575845
		R29W	1	56.797777, 59.426426
			2	56.798321, 59.427519
			3	56.799041, 59.427298
		R33W	1	56.806758, 59.362488
			2	56.807238, 59.362600
			3	56.808085, 59.360974
frame-designed pitfall trapping	spruce-fir forest litter	R20W-frame	–	56.821194, 59.57625

**Table 2.** Richness and abundance (adults/total) of species per habitat

Order	Family	Species	Central Urals			Southern Urals	Total
			Meadow grass stand	Spruce-fir forest litter	Aspen-birch forest litter	Pine-birch forest litter	
Araneae	Araneidae	*Araneus alsine*	2/2				2/2
		*Araneus marmoreus*	1/1				1/1
		*Araneus sturmi*	0/2				0/2
		*Cercidia prominens*	1/1			3/3	4/4
		*Cyclosa conica*	0/1				0/1
	Cheiracanthiidae	*Cheiracanthium erraticum*	2/2				2/2
	Clubionidae	*Clubiona caerulescens*	3/4	4/4		1/1	8/9
		*Clubiona kulczynskii*	7/7		1/1		8/8
		*Clubiona lutescens*	8/8		2/2		10/10
		*Clubiona neglecta*	1/1				1/1
		*Clubiona stagnatilis*	2/2				2/2
	Cybaeidae	*Cryphoeca silvicola*		21/21	4/4		25/25
	Dictynidae	*Dictyna arundinacea*	3/3	1/1			4/4
	Gnaphosidae	*Drassyllus pusillus*		1/1			1/1
		*Haplodrassus soerenseni*		6/6	1/1	27/27	34/34
		*Micaria pulicaria*	1/1				1/1
		*Zelotes clivicola*		2/2			2/2
		*Zelotes subterraneus*		2/2		9/9	11/11
	Hahniidae	*Antistea elegans*	4/4	1/1			5/5
		*Hahnia ononidum*		6/6	30/31		36/37
		*Hahnia pusilla*	3/3	30/30	1/1		34/34
	Linyphiidae	*Abacoproeces saltuum*				4/4	4/4
		*Abiskoa abiskoensis*		1/1			1/1
		*Agyneta affinis*	2/2		5/5		7/7
		*Agyneta allosubtilis*		5/5	2/2		7/7
		*Agyneta conigera*		36/36	2/2	1/1	39/39
		*Agyneta olivacea*		164/165	2/2	19/19	185/186
		*Agyneta ramosa*				11/11	11/11
		*Agyneta subtilis*			1/1		1/1
		*Allomengea scopigera*	1/1	2227/2229	284/284	3/3	2515/2517
		*Allomengea vidua*	13/14				13/14
		*Anguliphantes angulipalpis*		3/3	1/1	1/1	5/5
		*Asthenargus paganus*		175/175	5/5		180/180
		*Bathyphantes gracilis*		2/2	3/3		5/5
		*Bathyphantes nigrinus*	13/13	14/14	28/28		55/55
		*Bathyphantes parvulus*		10/10			10/10
		*Bolyphantes alticeps*	36/47	46/47	2/2	2/2	86/98
		*Centromerus arcanus*	1/1	150/150	3/3		154/154
		*Centromerus brevipalpus*				2/2	2/2
		*Centromerus clarus*		40/40		4/4	44/44
		*Centromerus levitarsis*	1/1				1/1
		*Centromerus sylvaticus*	2/2	338/338	20/20	3/3	363/363
		*Ceraticelus bulbosus*	4/4				4/4
		*Ceratinella brevipes*				5/5	5/5
		*Ceratinella brevis*	1/1	24/24	14/14		39/39
		*Ceratinella scabrosa*		8/8			8/8
		*Cnephalocotes obscurus*	3/3	1/1		1/1	5/5
		*Decipiphantes decipiens*		4/4			4/4
		*Dicymbium tibiale*		1/1	7/7		8/8
		*Diplocentria bidentata*		22/22			22/22
		*Diplocephalus picinus*		25/25	8/8	20/20	53/53
		*Diplostyla concolor*		9/10	2/2		11/12
		*Dismodicus bifrons*	9/9		1/1		10/10
		*Drapetisca socialis*		7/8			7/8
		*Erigonella hiemalis*		74/74	20/20		94/94
		*Erigonella ignobilis*	21/21		1/1		22/22
		*Flagelliphantes bergstromi*		1/1			1/1
		*Floronia bucculenta*	1/1	4/4	2/2		7/7
		*Gonatium rubellum*	1/1	10/10			11/11
		*Gongylidiellum latebricola*	1/1				1/1
		*Helophora insignis*		1/1		3/3	4/4
		*Hypselistes jacksoni*	5/5	10/10	3/3		18/18
		*Kaestneria pullata*	2/2				2/2
		*Leptorhoptrum robustum*		1/1			1/1
		*Linyphia triangularis*	1/1				1/1
		*Macrargus rufus*		33/33	12/12	6/6	51/51
		*Maro pansibiricus*		49/49	3/3	6/6	58/58
		*Maro sibiricus*		14/14		3/3	17/17
		*Metopobactrus prominulus*	6/6				6/6
		*Micrargus herbigradus*		5/5			5/5
		*Microlinyphia pusilla*	1/2				1/2
		*Microneta viaria*		116/116	34/34	25/25	175/175
		*Minyriolus pusillus*		30/30	1/1		31/31
		*Neriene clathrata*	5/5	3/3	1/1		9/9
		*Neriene emphana*	1/1	6/6	1/1		8/8
		*Neriene montana*		2/2	1/1		3/3
		*Notioscopus sarcinatus*	2/2				2/2
		*Obscuriphantes obscurus*		2/2			2/2
		*Oedothorax apicatus*			1/1		1/1
		*Oedothorax retusus*	2/2				2/2
		*Oryphantes geminus*		23/23	2/2		25/25
		*Palliduphantes alutacius*		31/31	4/4	1/1	36/36
		*Palliduphantes antroniensis*		1/1			1/1
		*Panamomops dybowskii*		7/7			7/7
		*Pocadicnemis pumila*	21/21		2/2	1/1	24/24
		*Porrhomma pallidum*		1/1			1/1
		*Semljicola faustus*		2/2			2/2
		*Semljicola thaleri*		46/46			46/46
		*Silometopus elegans*	1/1				1/1
		*Sintula corniger*			2/2		2/2
		*Stemonyphantes conspersus*		1/1			1/1
		*Styloctetor stativus*	6/6				6/6
		*Tallusia experta*	15/15		1/1		16/16
		*Tapinocyba insecta*		172/172	34/34	12/12	218/218
		*Tapinopa longidens*		6/6		1/1	7/7
		*Tenuiphantes alacris*	1/1	6/6			7/7
		*Tenuiphantes mengei*	1/1	1/1	6/6	8/8	16/16
		*Tenuiphantes nigriventris*		129/129	1/1	11/11	141/141
		*Tenuiphantes tenebricola*		136/136	40/40	17/17	193/193
		*Thyreosthenius parasiticus*			1/1		1/1
		*Tibioplus diversus*		11/11	1/1		12/12
		*Trematocephalus cristatus*	1/1	1/1			2/2
		*Walckenaeria alticeps*	3/3	1/1			4/4
		*Walckenaeria antica*		1/1	3/3	2/2	6/6
		*Walckenaeria atrotibialis*		18/18	7/7	5/5	30/30
		*Walckenaeria cucullata*		1/1			1/1
		*Walckenaeria dysderoides*				1/1	1/1
		*Walckenaeria mitrata*	1/1	1/1	5/5		7/7
		*Walckenaeria nodosa*		2/2			2/2
		*Walckenaeria nudipalpis*	1/1	62/62	14/14		77/77
		*Walckenaeria obtusa*		21/21			21/21
		*Walckenaeria unicornis*	2/2		1/1		3/3
		*Walckenaeria vigilax*	7/7				7/7
		*Zornella cultrigera*		7/7	1/1		8/8
	Liocranidae	*Agroeca brunnea*		6/6		2/2	8/8
		*Agroeca proxima*		2/2	3/3	23/23	28/28
	Lycosidae	*Alopecosa aculeata*				30/30	30/30
		*Alopecosa pinetorum*		2/2			2/2
		*Alopecosa pulverulenta*		1/1			1/1
		*Alopecosa sulzeri*				2/2	2/2
		*Alopecosa taeniata*		14/14	4/4	143/143	161/161
		*Pardosa amentata*			1/1		1/1
		*Pardosa fulvipes*	19/21				19/21
		*Pardosa lugubris*	7/7	45/45	63/63	74/74	189/189
		*Pardosa riparia*	1/1	1/1			2/2
		*Pardosa sphagnicola*	7/8	3/3			10/11
		*Pirata uliginosus*	6/6	1/1	1/1		8/8
		*Piratula hygrophila*	21/24	51/54	454/532	2/2	528/612
		*Trochosa ruricola*	5/5	1/1			6/6
		*Trochosa spinipalpis*		2/2			2/2
		*Trochosa terricola*	2/2	8/8	20/20	10/10	40/40
		*Xerolycosa nemoralis*			0/2		0/2
	Mimetidae	*Ero cambridgei*	1/1				1/1
		*Ero furcata*		16/16	1/1	1/1	18/18
	Miturgidae	*Zora nemoralis*				26/26	26/26
		*Zora spinimana*	4/5	4/4	2/2	5/6	15/17
	Oxyopidae	*Oxyopes ramosus*	0/24				0/24
	Philodromidae	*Thanatus sabulosus*				3/3	3/3
		*Tibellus oblongus*	1/3				1/3
	Phrurolithidae	*Phrurolithus festivus*				1/1	1/1
	Pisauridae	*Dolomedes fimbriatus*	2/2				2/2
		*Pisaura mirabilis*	0/6				0/6
	Salticidae	*Dendryphantes rudis*		1/1			1/1
		*Euophrys frontalis*	3/5				3/5
		*Evarcha arcuata*	1/1				1/1
		*Evarcha falcata*	12/13			3/3	15/16
		*Marpissa pomatia*	16/26				16/26
		*Sibianor larae*	3/3				3/3
		*Talavera aequipes*	1/1				1/1
	Sparassidae	*Micrommata virescens*	1/16				1/16
	Tetragnathidae	*Metellina mengei*		4/4	2/2		6/6
		*Pachygnatha degeeri*	1/1	1/1			2/2
		*Pachygnatha listeri*	7/8	20/20	13/13	1/1	41/42
		*Tetragnatha extensa*	1/1				1/1
		*Tetragnatha pinicola*	4/4				4/4
	Theridiidae	*Canalidion montanum*			1/1		1/1
		*Cryptachaea riparia*	1/1				1/1
		*Enoplognatha ovata*	1/2				1/2
		*Neottiura bimaculata*	8/8				8/8
		*Robertus lividus*		54/55	11/11	14/14	79/80
		*Rugathodes aurantius*	13/13				13/13
		*Theridion varians*				2/2	2/2
	Thomisidae	*Misumena vatia*	2/7				2/7
		*Ozyptila praticola*		1/1		37/37	38/38
		*Ozyptila trux*	10/10	10/10	4/4	2/2	26/26
		*Xysticus audax*		9/9	2/2		11/11
		*Xysticus cristatus*		5/5			5/5
		*Xysticus lanio*				1/1	1/1
		*Xysticus luctuosus*	2/2			47/47	49/49
		*Xysticus obscurus*		1/1			1/1
		*Xysticus ulmi*	1/1				1/1
Opiliones	Phalangiidae	*Lacinius ephippiatus*	9/28	212/548	35/108	0/1	256/685
		*Lophopilio palpinalis*	14/36	20/40			34/76
		*Mitopus morio*	6/6	55/115	6/242		67/363
		*Oligolophus tridens*	99/152	229/437	28/42		356/631
		*Phalangium opilio*	2/3				2/3
		*Rilaena triangularis*	6/11	3/81	0/25		9/117
	Nemastomatidae	*Nemastoma lugubre*	11/14	838/846	96/96	5/5	950/961
	**Total species**		**92**	**106**	**74**	**55**	
	**Species abundance**		**738**	**6775**	**1810**	**654**	
	**Total abundance**		**752**	**7091**	**1816**	**774**	

**Table 3.** Habitat differentiation in age-sex composition of the Arachnida families (sex status for the adult and undamaged specimens only)

Family	Meadow grass stand			Spruce-fir forest litter			Aspen-birch forest litter			Pine-birch forest litter			**Total**
	Adult		Juvenile	Adult		Juvenile	Adult		Juvenile	Adult		Juvenile	
	Male	Female		Male	Female		Male	Female		Male	Female		
Araneidae	2	2	3			7					3	1	**18**
Cheiracanthiidae	1	1											**2**
Clubionidae	18	3	1	1	3	1		3		1			**31**
Cybaeidae				2	19			4					**25**
Dictynidae	3				1								**4**
Gnaphosidae	1			3	8			1		8	28	2	**51**
Hahniidae	5	2		1	36		3	28	1				**76**
Linyphiidae	149	46	13	1484	2931	158	204	395	2	61	128	30	**5601**
Liocranidae			1	6	2			3		6	19	2	**39**
Lycosidae	62	6	9	32	98	21	195	348	80	43	218	51	**1163**
Mimetidae	1			3	13	5	1				1		**24**
Miturgidae	3	1	1		4			2		4	27	2	**44**
Oxyopidae			24										**24**
Philodromidae	1		9								3		**13**
Phrurolithidae											1		**1**
Pisauridae	2		6										**8**
Salticidae	23	13	13	1		1				3	1		**55**
Sparassidae	1		15										**16**
Tetragnathidae	6	7	4	13	12	1	8	7		1			**59**
Theridiidae	22	1	1	9	45	1	2	10		5	11		**107**
Thomisidae	13	2	5	3	23	9	1	5		17	70	13	**161**
Phalangiidae	90	46	100	273	246	764	36	33	348			9	**1945**
Nemastomatidae	6	5	3	438	399	8	52	44			5		**960**
**Total**	**409**	**135**	**208**	**2269**	**3840**	**976**	**502**	**883**	**431**	**149**	**515**	**110**	
